# Effect of point of care blood testing on physical health check completion in mental health services: mixed-methods evaluation

**DOI:** 10.1192/bjo.2020.110

**Published:** 2020-10-27

**Authors:** Joseph Butler, Simone de Cassan, Margaret Glogowska, Thomas R. Fanshawe, Phil Turner, Debbie Walton, Daniel Lasserson, Robert Bale, Belinda Lennox, Gail Hayward

**Affiliations:** Department of Psychiatry, University of Oxford, UK; Oxford Health NHS Foundation Trust, UK; Nuffield Department of Primary Care Health Sciences, University of Oxford, UK; Nuffield Department of Primary Care Health Sciences, University of Oxford, UK; National Institute for Health Research Community Healthcare Healthcare MedTech and In Vitro Diagnostics Co-operative, Nuffield Department of Primary Care Health Sciences, University of Oxford, UK; Adult and Older Adult Mental Health, Oxford Health NHS Foundation Trust, UK; Institute of Applied Health Research, University of Birmingham; and Department of Acute Medicine, City Hospital, Sandwell and West Birmingham Hospitals NHS Trust, UK; Adult Mental Health Services, Oxford Health NHS Foundation Trust, UK; Oxford Health NHS Foundation Trust; Department of Psychiatry, University of Oxford, UK; National Institute for Health Research Community Healthcare MedTech and In Vitro Diagnostics Co-operative, Nuffield Department of Primary Care Health Sciences, University of Oxford, UK

**Keywords:** Psychotic disorders, qualitative research, community mental health teams, outcome studies, comorbidity

## Abstract

**Background:**

Physical health outcomes in severe mental illness are worse than in the general population. Routine physical health check completion in this group is poor.

**Aims:**

To quantitatively and qualitatively evaluate the impact of point of care (POC) blood testing on physical health check completion in community mental health services.

**Method:**

In a prospective cohort design, we equipped an early intervention service (EIS) and a community mental health team (CMHT) with a POC blood testing device for 6 months. We compared rates of blood test and full physical health check completion in the intervention teams with a matched EIS and CMHT, historically and during the intervention. We explored attitudes to POC testing using thematic analysis of semi-structured interviews with patients and clinicians.

**Results:**

Although the CMHT scarcely used the POC device and saw no change in outcomes, direct comparison of testing rates in the intervention period showed increased physical health check completion in the EIS with the device (rate ratio RR = 5.18; 95% CI 2.54–12.44; *P* < 0.001) compared with usual care. The rate was consistent with the EIS's increasing rate of testing over time (RR = 0.45; 95% 0.09–2.08; *P* = 0.32). Similar trends were seen in blood test completion. POC testing was acceptable to patients but clinicians reported usability, provision and impact on the therapeutic relationship as barriers to uptake.

**Conclusions:**

POC testing was beneficial and acceptable to patients and may increase physical health check uptake. Further research, accounting for clinician barriers, is needed to evaluate its clinical and cost-effectiveness.

People with severe mental illnesses such as schizophrenia and bipolar disorder have a life expectancy 15–20 years below the general population.^1–3^ This mortality gap is mainly caused by comorbid physical illness, particularly preventable cardiovascular complications.^4^ People with severe mental illness experience additional cardiovascular risk through the effects of antipsychotic medication,^5^ chronic stress,^6^ unhealthy lifestyle behaviours^7^ and diagnostic overshadowing and symptom misattribution.^8^ Mental illness, socioeconomic status and multimorbidity are strongly associated^9^ and there is evidence of shared genetic risk factors.^10^

The National Institute for Health and Care Excellence (NICE) guideline on psychosis and schizophrenia^11^ recommends monitoring physical health, and a physical health check is a NICE quality standard of care for early psychosis. The check comprises a targeted history and examination, assessing the patient's lifestyle behaviours (diet and exercise), use of substances, blood pressure, body mass index, glycated haemoglobin (HbA_1c_) and lipid panel blood tests and estimating the risk of developing heart attack or stroke over the next decade using the QRisk calculator.^12^ NICE endorses these investigations to identify those who may benefit from targeted lifestyle advice, antihypertensive, metformin or lipid modification therapy, or referral to specialist services.^13^ Mental health services are expected to provide this check annually, but completion is poor. Only 32.3% of patients with severe mental illness receive a full check, with blood tests the component most commonly missed.^14^

A complete health check requires multiple appointments, but patients might be missing out. Around 20% of psychiatry out-patient appointments are not attended,^15^ and there are barriers to accessing physical healthcare owing to stigma, level of clinician training, difficulties communicating and ‘navigating the system’, symptoms of mental illness^16^ and ethnic background.^17^

Point of care (POC) lipid panel and HbA_1c_ tests could increase screening rates.^17^ They require minimal expertise, are run at or near the site of the patient, typically use ‘pin-prick’ samples of blood and give results within minutes. This would allow full physical health checks to be carried out a convenient time and place, in one visit, by a patient's mental healthcare provider. We implemented POC HbA_1c_ and lipid testing in an early intervention service (EIS) and a community mental health team (CMHT) over a 6-month period. We compared EISs and CMHTs that had access to POC testing with those that did not, measuring the numbers of blood tests and health checks completed. We conducted qualitative interviews with clinicians who were using the POC devices and patients whose care was augmented with POC testing. This is the first time POC blood testing has been evaluated in psychiatry.

## Method

### Ethical approval

Ethical approval for the qualitative arm of the study was provided by the subcommittee of Wales Research Ethics Committee 6 (reference: 18/WA/0302). The quantitative evaluation using routine data collection by the direct care team was prospectively approved as a service evaluation by Oxford Health NHS Foundation Trust.

### Study design

A prospective cohort design compared two CMHTs in Oxfordshire and two EISs (one in Oxfordshire and one in Buckinghamshire). The Oxfordshire EIS serves a population of 687 524 and was given a POC device for the study period (mid-November 2018 to mid-May 2019). The Buckinghamshire EIS, which serves a population of 540 059, was not given access to POC testing. Both CMHTs served the South Oxfordshire area (population = 140 504). The CMHT receiving a POC device in the study period (mid-December 2018 to mid-June 2019) had around 450 patients on its case-load, and the team without a POC device had a case-load of around 290. We collected data on screening rates during the study period and across three previous years (August 2015 to August 2016, August 2016 to August 2017 and August 2017 to August 2018) for historical comparison. Qualitative data on clinician and patient experience and perceptions of POC testing were also collected during the study period.

### Patient eligibility

Early intervention services provide support during the first 3 years of psychotic illness for patients aged 14–65. All patients on EIS case-loads are eligible for a yearly physical health check. CMHTs provide support for people aged 18+ with a range of mental health problems. Individuals with severe mental illness (defined as schizophrenia, bipolar affective disorder and other psychoses)^18^ are eligible for a yearly physical health check. If patients were eligible, they were offered POC testing by their clinical team either in a clinic or in the patient's home.

Patients were excluded from data collection if they did not qualify for a yearly physical health check, they had not been in the service for the full duration of the period being analysed or they had had an in-patient admission during the period of analysis.

### Point of care (POC) testing device and training

The Afinion™ 2 analyser (Abbott Healthcare) was used for lipid panel and HbA_1c_ testing, following local laboratory validation.

A number of training sessions were organised with the clinical teams and educational materials were provided to help with interpretation of results and troubleshooting for the device. A clinical care pathway was developed to alert the medical team and subsequently the patient's general practitioner (GP) of any abnormal results. Clinicians trained to use the device were given a log-in number to it. Clinicians operating the device transcribed results into the patients’ electronic health records (EHRs). At each team's ‘base’, an ethernet port was activated to allow for upload of results to the biochemistry laboratory at the John Radcliffe Hospital, Oxford.

### Quantitative data collection

All cohorts were tested at four separate time points: pre-intervention in August 2016, 2017 and 2018, and post-intervention in May 2019 (for the EIS) and June 2019 (for the CMHT) over the study period. For each time point, a list of patients on the case-load of each team was acquired.

The EHRs of the eligible patients were audited and demographic data, including gender, age, time with the service, diagnosis and medications, were recorded. Researchers gathered data over the previous year (pre-intervention cohort) or previous 6 months (post-intervention cohort). Outcome measures included whether patients had an HbA_1c_ or lipid panel test recorded, along with the date, location, result, whether any action had been initiated and how long until that action had taken place. It was also noted whether the other components of the physical health check (smoking status, alcohol use, body mass index, blood pressure, diet and exercise status) had been recorded.

### Data analysis

Between-group comparisons of intervention and control cohorts were made in two ways. First, the 6-monthly post-intervention rates of testing were compared directly (unadjusted) between each intervention cohort and the corresponding control cohort. Second, this comparison was made adjusting for the historical time trend for completion at each site, using data from three previous time points (2015–2016, 2016–2017 and 2017–2018). This comparison can be interpreted as the additional effect of intervention versus control after allowing for this historical trend.

Analyses were performed using Poisson regression with an offset to allow for differences in follow-up times between pre-intervention and post-intervention time points (i.e. 12-monthly data for pre-intervention and 6-monthly for post-intervention). The model included site-specific intercept and linear gradient terms (to allow for differential trends between sites), with indicator variables representing the effect of the post-intervention period additional to the historical trend. Rates calculated on the basis of data from 12-month periods are presented on a ‘per 6-month’ basis to allow direct comparison between time points. Effect sizes are expressed as rate ratios (RR) with 95% confidence intervals (CI) and *P*-values.

All analyses were performed separately for each team type, i.e. EIS or CMHT. Three outcomes are presented: screening rates for HbA_1c_, for lipid panel and for all eight available tests.

### Qualitative study

We aimed to explore both patients’ and clinicians’ views of POC testing, through semi-structured interviews. These were conducted by J.B. and S.d.C. in parallel with quantitative data collection.

Patients who had POC testing during the study period were approached by their primary mental health clinician for consent for the research team to contact them about the study. Fourteen patient interviews were conducted: nine over the phone and five face to face. S.d.C. and J.B. used a topic guide to explore perceptions and experiences of care augmented with POC testing. An initial patient topic guide was developed from the literature and using the experience of our research team. This guide was adapted as topics evolved and emerged during the interviews.

Clinicians who had access to POC results were also approached for involvement. Fifteen clinicians were interviewed, both face-to-face (11) and over the telephone (4). A clinician topic guide was developed and used flexibly by S.d.C. and J.B. to conduct semi-structured interviews, similarly to patient data collection described above.

Interviews were audio-recorded and transcribed verbatim. Interviews continued until no new themes were emerging and there was sufficient explanation of those themes. Transcripts were analysed using Windows NVivo 11 (QSR International) using principles of thematic analysis.^19^

All participants gave written informed consent for participation in the study and for publication of the project findings and written quotations.

### Data analysis

Patient and clinician data were analysed separately. Data analysis was guided by the constant comparative method, which involves reading of the transcripts, noting and recording initial themes and then applying systematic and detailed open coding using NVivo 11.^20^ The coding framework was derived from the topic guide and refined after initial double coding of transcripts by S.d.C. and J.B. as well as discussions among the whole research team. The research team took an iterative stance combining early analysis with ongoing data collection. This allowed for the inclusion of emerging categories from the data and ensured that themes and concepts were grounded in the data.

## Results

Baseline demographics for the teams’ case-loads during the intervention periods are shown in Table 1. Table 2 shows the numbers of clinicians trained in using POC testing at the intervention sites.

**Table 1:** Baseline demographics of the teams’ case-loads

	CMHT with POC	CMHT with usual care	EIS with POC	EIS with usual care
Patients, *n*	158	86	149	90
Male, *n* (%)	95 (60)	46 (53)	89 (60)	55 (61)
Age, years: mean (s.d.)	45 (11.79)	43 (10.31)	32 (12.19)	31 (12.06)

CMHT, community mental health team; POC, point of care blood testing; EIS, early intervention service.

**Table 2:** Clinicians at the intervention sites trained in point of care (POC) blood testing

Clinician's profession	CMHT clinicians with POC training, *n*	EIS clinicians with POC training, *n*
Community psychiatric nurse	9	8
Social worker	11	7
Psychologist	0	2
Occupational therapist	0	1
Total	20	18

CMHT, community mental health team; EIS, early intervention service.

### Quantitative outcome measures

**Fig. 1:**
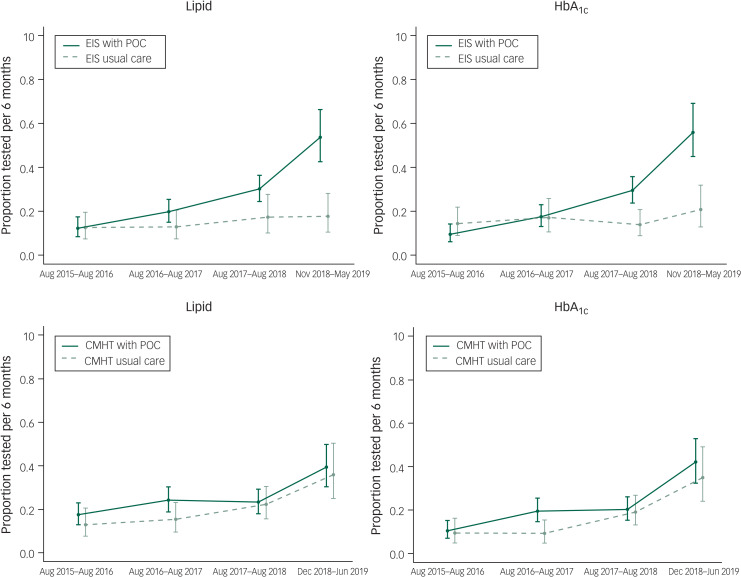
Rates of blood test completion for teams provided with point of care (POC) blood testing devices compared with those giving care as usual. Data are shown for the study period and three previous years. Rates calculated using data from the previous years are presented on a ‘per 6-month’ basis to allow direct comparison between time points. EIS, early intervention service; CMHT, community mental health team.

Rates of completion of blood tests are shown in Table 3 and Fig. 1. A similar relationship was seen for both HbA_1c_ and lipid panel testing. For the EIS teams, direct comparison between the intervention group and the control group revealed a significant increase in the rate of blood test completion (lipid panel: RR = 3.02, *P* < 0.001; HbA_1c_: RR = 2.67, *P* < 0.001). The team using POC testing, however, had higher historical rates of completion and when this trend was accounted for, the effect diminished (lipid panel: RR = 1.29, *P* = 0.42; HbA_1c_: RR = 0.88, *P* = 0.76). No differences were seen between the CMHTs.

**Table 3:** Completion of blood tests and full physical health checks by the intervention and control teams

Team	Metric	Estimated 6-month rate of testing in intervention period, % (95% CI)	Direct comparison of intervention rates, RR^a^ (95% CI)	Adjusted comparison of intervention rates, RR^a^ (95% CI)
EIS with POC	Lipid panel	53.7 (42.8–66.3)	3.02 (1.82–5.36), *P* < 0.001	1.29 (0.61–3.27), *P* = 0.42
EIS usual care		17.8 (10.4–28.0)		
CMHT with POC		39.2 (30.3–49.8)	1.09 (0.71–1.70), *P* = 0.70	1.09 (0.52–2.28), *P* = 0.82
CMHT usual care		36.0 (24.8–50.3)		
EIS with POC	HbA_1c_	56.4 (45.2–69.3)	2.67 (1.66–4.52), *P* < 0.001	0.88 (0.38–2.01), *P* = 0.76
EIS usual care		21.1 (13.0–32.1)		
CMHT with POC		41.8 (32.5–52.7)	1.20 (0.79–1.87), *P* = 0.41	1.08 (0.47–2.46), *P* = 0.85
CMHT usual care		34.9 (23.8–48.9)		
EIS with POC	Full physical health check	40.3 (30.9–51.3)	5.18 (2.54–12.44), *P* < 0.001	0.45 (0.09–2.08), *P* = 0.32
EIS usual care		7.8 (3.3–15.0)		
CMHT with POC		8.2 (4.5–13.5)	1.77 (0.63–6.28), *P* = 0.32	–^b^
CMHT usual care		4.7 (1.4–10.8)		

EIS, early intervention service; POC, point of care blood testing; CMHT, community mental health team.

a. The rate ratio (RR) shows the ratio of the estimated 6-month rate of testing for the POC teams to the estimated 6-month rate of testing for the usual care teams.

b. Pre-intervention rates were too low (including some zero rates) to allow model fitting.

**Fig. 2:**
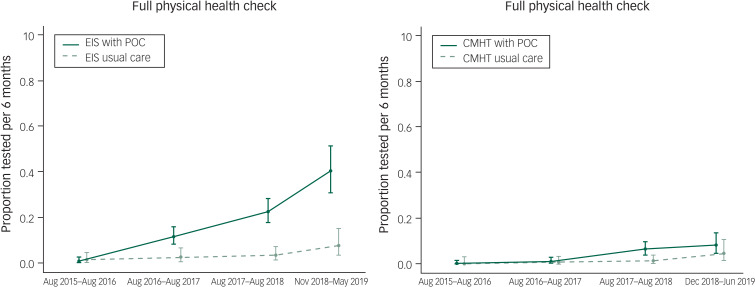
Rates of full physical health check completion for teams provided with point of care (POC) blood testing devices compared with those giving care as usual. Data are shown for the study period and three previous years. Rates calculated using data from the previous years are presented on a ‘per 6-month’ basis to allow direct comparison between time points. EIS, early intervention service; CMHT, community mental health team.

A similar outcome was seen when rates of full physical health check completion were analysed (Table 3 and Fig. 2). When comparing directly, there was a significant effect of the POC intervention in the EIS teams (RR = 5.18, *P* < 0.001), but this reduced and became non-significant when adjusting for an increasing historical completion rate (RR = 0.45, *P* = 0.32). In all teams other than the EIS using POC testing, physical health check completion did not exceed 10% of patients.

### Clinician qualitative findings

We conducted 15 interviews with clinicians in the intervention teams: 11 from the EIS and 4 from the CMHT. Of the interviewees, six were from a psychiatric nursing background, three were medical professionals and six were allied health professionals. The following three themes emerged that highlight general barriers to POC utilisation, as well as specific views across the two teams that could explain the difference in POC testing uptake.

#### Theme 1: Views on physical healthcare provision

There was a lack of clarity about whether mental health teams or GPs should be responsible for patients’ physical health, including monitoring:

‘I don't think, and [GPs] tend to agree, … that it's a good use of our time to be doing ECGs and bloods. They're comfortable with doing them if it means we can get on more with doing mental health stuff…’ (C012, Medical, CMHT)

‘I mean, I know that the policy is that we should be doing it [physical health screening], but I personally think that people need to go to the GP. I just think it's normal.’ (C008, Allied Health, EIS)

There was also a feeling among clinicians that additional work would add to the pressures already faced by services:

‘My first impression was like, “Oh no, that's another thing,” and it added work for us to do, and then I thought, “Oh no, I don't think I'll be able to use it”.’ (C013, Nursing, EIS)

‘You've got so many competing priorities … and physical health is often the one that slips off.’ (C009, Medical, EIS)

#### Theme 2: Ease of use of the POC device

Clinician engagement with POC testing was affected by practical difficulties in use of the device. This included concern over damaging the expensive equipment, patient's home environments being unsuitable and high failure rates of the analyser's cartridges:

‘… some of the patients we see it's difficult to find a surface even and a plug that you can plug into.’ (C004, Nursing, CMHT)

All clinicians experienced errors and mistakes: those that used the device more tended to embody the adage ‘practice makes perfect’:

‘… the more you do it the better you get at it … Before when I first started using it, it used to take me like an hour, twenty minutes to do it. And now you're just taking thirty minutes to do it.’ (C013, Nursing, EIS)

Members of the CMHT felt that the high failure rate of the device made them reconsider using it for physical health monitoring:

‘We gave up doing the lipids in the end because every time we had problems with it unless you guys were there …’ (C015, Allied Health, CMHT)

#### Theme 3: Impact of POC testing on the therapeutic relationship

Concerns about appearing unprofessional in front of patients if there was an error with the machine were highlighted by clinicians as a reason for non-engagement with POC testing:

‘… it just seemed a little bit unprofessional when I was in somebody's house that was quite willing to participate, and then I couldn't complete the whole thing.’ (C010, Nursing, EIS)

‘… there were too many errors that often would leave the patient waiting, then just have to say, you know, we couldn't leave them waiting that long. So we didn't get on so well with it.’ (C015, Allied Health, CMHT)

### Patient qualitative findings

We interviewed 14 patients (average age 33 years (range 19–66), 10 male, 4 female) to explore acceptability of POC testing to patients. The following three themes became apparent relating to patient experience of care augmented with POC testing.

#### Theme 1: Anxiety

Most patients had no knowledge of POC testing and experienced anxiety and curiosity about the prospect of receiving it. When anxiety occurred, it diminished after testing:

‘More anxious going in than coming out’ (P003)

Patients compared POC testing with the traditional blood test pathway, finding it less anxiety-provoking:

‘I turned [the GP] down because of the anxiety for going, so this was the only way really I could have the test …’ (P012)

POC testing helped patients feel reassured:

‘It makes me feel more secure. I'm less worried now; I don't feel that I need another health check for a while.’ (P008)

‘But the good thing about having that like physical health check is to make sure I've not got [diabetes] … But I don't have it, which is good.’ (P010)

#### Theme 2: Efficiency

Patients enjoyed the intrinsic benefits of POC testing, as well as how care it seemed more efficient than the traditional testing pathway:

‘… just recommend anybody to have it done and it's extremely easy and quick and painless.’ (P014)

‘… this was just one journey and, you know, as you know the results there and then, so it was sort of very quick and very concise, yeh.’ (P003)

Several patients highlighted that POC testing provided a less invasive alternative when they had difficult veins or were needle phobic:

‘Because like usually when the GP gets my blood tests done they like have to bring other nurses in to do it because they can't find my vein properly.’ (P010)

#### Theme 3: Engagement with physical health

Accessing results at the point of contact with their clinician was desirable; discussions could be focused on specific concerns, making results more meaningful:

‘… it's handy because all of the questions that I had in my mind I could ask then and I could, you know, get professional opinion on it, whereas if they were like sent to me as a text I'd just look at some numbers and I wouldn't really … I wouldn't really register it.’ (P002)

Patients reported an enhanced understanding of their physical health and appreciated the opportunity to obtain answers to their questions about their health:

‘It made me understand a bit more about blood and what levels are good and what levels aren't good, as in like diabetes, what the threshold is and what if it's above a certain threshold then you're going closer to diabetes.’ (P004)

## Discussion

### Summary of findings

This study found that in an EIS team given a POC blood testing device for physical health check completion, over 50% of the eligible case-load received an HbA_1c_ and lipid panel test over a 6-month intervention period. This is consistent with the entire case-load being tested every 12 months. In the same period, a comparison EIS team without POC testing had lower rates of blood test completion.

There was therefore an increase in the rate of physical health check completion in the EIS team with POC testing available. However, this team had also seen an increasing rate of health check completion in the 3 years prior to the POC testing pilot, so the rate of increase in testing seen in this team was not statistically significant.

POC testing had limited uptake in the CMHT, with no effects on rates of blood test and physical health check completion.

Qualitative analysis of participating clinicians’ interviews helps to explain the varied uptake across the two teams. Different attitudes regarding the mental healthcare remit, difficulty using the testing device and concern about impact on the therapeutic relationship appeared to underpin clinicians’ decisions to not use the device. Support and perseverance may help overcome these barriers. In contrast to clinician reluctance, patients in our sample were enthusiastic about the benefits of POC testing, preferring the less invasive and more efficient testing, and they reported increased understanding and engagement with their physical health. However, our findings are based on a small number of participants.

### Comparison with the literature

To our knowledge, this is the first evaluation of POC blood testing in community mental health services. POC testing has been used in primary care, to help urgent clinical decisions such as whether to admit a patient to hospital^21^ or whether to prescribe antibiotics,^22^ and it has been argued that it is cost-effective in providing physical health checks in primary care.^23^ In these contexts, clinicians have found that POC testing helps them empower and educate their patients.^24^

Implementation of POC testing has been hindered by difficulties using the device, and constraints on clinician time and resources.^21^ There is evidence that barriers can be overcome if clinicians perceive value in the device.^25^ Although these themes emerged in our analysis, our clinicians reported the additional barriers of physical healthcare being seen as outside of their remit and of POC testing as having a negative impact on the therapeutic relationship. This might reflect the fact that the majority of research on POC testing focuses on primary care, where clinicians manage all aspects of patients’ health and have prior expertise in POC test interpretation and communication of results.

### Strengths and limitations

This was a real-world study, undertaken in National Health Service (NHS) EISs and CMHTs and was inclusive of all those on the clinical case-load, rather than a selected number recruited to a research study. The number of sites was limited, and community mental health services' obligations for physical health check provision vary, with varying contractual arrangements between these services, primary care and laboratories across the country. We were not able to assess the effect of patient ethnicity on the uptake of testing, due to the demographics of the region. Furthermore, there were increasing rates of health check completion in the EIS team provided with the POC testing device prior to the introduction of the device, suggesting that the team was already more engaged in providing physical healthcare for their patients. Therefore there may be other reasons why other teams did not engage with physical healthcare, such as team structure, systemic problems, or contracting arrangements that were not measured in this study.

The intervention period was 6 months, whereas the care standard for patients in these services is an annual physical health check. We have assumed that the rate of health check completion does not vary across a year.

### Implications for practice

Preliminary data show that POC blood testing was preferred by patients and may increase physical health check completion. A key modifiable barrier to implementation is the feeling among CMHT and EIS staff that physical healthcare is not part of their job. Clear expectations of their role in undertaking physical health checks is therefore needed to improve implementation, and this should be from both commissioners and providers of mental healthcare.

Further study, particularly randomised controlled trials, is needed to evaluate the clinical and cost-effectiveness of POC testing technology in severe mental illness.

## Data Availability

Anonymised and de-identified study data may be requested from the corresponding author. Study data are not publicly available owing to concerns that participant privacy may be compromised.
